# Role of Surgery for Pancreatic Ductal Adenocarcinoma in the Era of Multidisciplinary Treatment

**DOI:** 10.3390/jcm12020465

**Published:** 2023-01-06

**Authors:** Kosei Takagi, Yuzo Umeda, Ryuichi Yoshida, Tomokazu Fuji, Kazuya Yasui, Takahito Yagi, Toshiyoshi Fujiwara

**Affiliations:** Department of Gastroenterological Surgery, Okayama University Graduate School of Medicine, Dentistry, and Pharmaceutical Sciences, Okayama 700-8558, Japan

The incidence and mortality rates of pancreatic ductal adenocarcinoma (PDAC) have increased in recent years worldwide [1]. Recently, there have been remarkable improvements in multidisciplinary treatment for PDAC; however, the prognosis for this disease remains poor, with a 5-year overall survival rate of approximately 10% [2]. Despite the current developments of multidisciplinary treatment for PDAC, surgery is regarded as the only treatment with curative potential [3].

Gemcitabine mono-therapy showed a survival benefit as a first-line therapy for patients with advanced PDAC or as adjuvant therapy for patients with resected PDAC [4,5]. Over time, gemcitabine-based combination therapies, including nab-paclitaxel or capecitabine plus gemcitabine, have demonstrated increased survival in the adjuvant and metastatic setting [6,7]. Currently, paradigms have shifted to a potentially more effective regimen such as FOLFIRINOX, resulting in longer survival [8,9,10]. Additionally, the use of neoadjuvant therapy in resectable and borderline resectable PDAC has increased [11,12]. Thus, with these recent developments of neoadjuvant and adjuvant therapies for PDAC, the role of surgery for PDAC has changed.

Standard resection types for PDAC include pancreatoduodenectomy with or without vascular resections, distal pancreatectomy, and total pancreatectomy. Regarding the role of vascular resection for PDAC, a recent meta-analysis has shown the safety and feasibility of venous resection during pancreatic surgery with comparable short- and long-term outcomes [13]. Extended lymphadenectomy in pancreatoduodenectomy did not have survival benefits [14]. The current trend has shifted to achieving R0 resection rather than performing extended lymphadenectomy in patients with PDAC.

As minimally invasive surgery, including laparoscopic and robotic surgeries, has developed in the field of gastrointestinal surgery, minimally invasive pancreas resection (MIPR) has become mainstream in the past decade [15]. However, the Miami International Evidence-based Guidelines on MIPR have shown weak evidence regarding MIPR for PDAC due to a lack of high-quality data [15]. Thus, further research is required, even though MIPR on PDAC might be safe and feasible. Furthermore, the impact of MIPR for PDAC after neoadjuvant therapy remains questionable.

Considering the growing evidence of MIPR and the current trends in the transition from open to minimally invasive surgery, the indications of MIPR for PDAC will increase. Moreover, MIPR using the robotic platform will be expanded due to the complexity of pancreatic surgery [16]. Therefore, it has become crucial to precisely understand the anatomical landmarks during MIPR. The superior mesenteric artery (SMA) is an important landmark in MIPR, including pancreatoduodenectomy and distal pancreatectomy, especially for PDAC. Although various approaches to the SMA during MIPR have been reported [17,18], it is important to establish robot-specific surgical strategies in performing robotic surgery.

In robotic pancreatoduodenectomy for PDAC, we proposed our surgical protocol and strategies for dissecting around the SMA [19,20]. In robotic distal pancreatectomy for PDAC, we reported a radical antegrade modular pancreatosplenectomy (RAMPS) technique using the supracolic anterior SMA approach [21]. The splenic artery is also an important anatomical landmark in distal pancreatectomy [22]. Accordingly, we should understand various surgical approaches and select the most suitable approach to perform robotic pancreatectomy safely.

The current Special Issue, “Current Surgical Management of Pancreatic Cancer,” in the Journal of *Clinical Medicine*, is dedicated to collecting high-quality scientific articles that focus on open and minimally invasive surgery, neoadjuvant and adjuvant therapies, and the outcomes after surgery for PDAC. Moreover, multimedia manuscripts for MIPR, including surgical techniques, strategies, and outcomes, are welcomed.

As the Guest Editors, we would like to sincerely appreciate and thank the reviewers for their insightful feedback and the JCM team’s support. In addition, we heartily thank all of the authors for their valuable input.
